# MITF Is an Essential and Functionally Multifaceted Transcription Factor in Cutaneous Melanoma

**DOI:** 10.3390/cancers18132160

**Published:** 2026-07-06

**Authors:** Lubica Ondrušová, Kateřina Kreisingerová, Jiri Vachtenheim

**Affiliations:** Department of Transcription and Cell Signaling, Institute of Medical Biochemistry and Laboratory Diagnostics, First Faculty of Medicine, General University Hospital, Charles University, 12108 Prague, Czech Republic; lubica.ondrusova@gmail.com (L.O.); vlckova.katka@centrum.cz (K.K.)

**Keywords:** melanoma, MITF, lineage identity, pro-survival factor, transcriptional circuitry, oncogene, apoptosis

## Abstract

Melanoma incidence is steadily on the rise but widespread prevention awareness and novel treatment approaches have substantially ameliorated the prognosis of the disease. Microphthalmia-associated transcription factor (MITF) is an essential transcription factor that plays a central role in the transcriptional circuitry of both normal melanocytes and malignant melanoma. Since over 30 years have elapsed since its discovery in mice, a large number of its target genes have been identified in pigment cells. Many upstream regulators of MITF have also been identified. Despite these substantial discoveries, MITF function, especially in melanomas, still remains elusive in several aspects. MITF is absolutely required for melanin formation because it transcribes virtually all genes required for the synthesis, storage, and transport of the pigment. Importantly, MITF is necessary for prevention of apoptosis in melanomas, at least at the early stages. However, in some metastases, MITF may be absent in most cells and its antiapoptotic function is evidently replaced by other proteins that not yet been fully identified. Furthermore, MITF is a specific nevus and melanoma marker, which is routinely used in immunohistochemistry, along with other markers, to distinguish pigmented and other skin lesions. In melanomas, high-MITF melanoma cell subpopulations are considered differentiated, i.e., pigmented and rapidly proliferating. In contrast, low-MITF cells proliferate slowly but are invasive with cancer stem cell-like properties. Although MITF activates mostly antiapoptotic and pro-proliferative genes, it also activates typical cell cycle inhibitors such as the p16 and p21 proteins. Here we discuss the issues of MITF multifunctionality in melanoma and associated research prospects.

## Simple Summary

Human malignant melanoma originates from neural crest-derived melanocytes. It is a highly aggressive and drug-resistant tumor characterized by the expression of the melanocyte-specific isoform of the transcription factor (MITF), which is a lineage-determining protein required for the differentiation phenotype of pigment cells. However, MITF also has pro-oncogenic activity mainly because it upregulates the expression of several antiapoptotic genes. Melanoma is a highly heterogenous tumor and contains populations with high and low MITF expression that behave differently during tumor progression.

## 1. Introduction

The microphthalmia-associated transcription factor (MITF) holds a pivotal function in the melanoma transcriptional machinery as it activates dozens of melanocyte-specific genes. During embryonic development, melanocytes evolve from neural crest precursor cells and then migrate to the epidermis [1,2,3]. The Sox2 protein, together with MITF, is also important for melanocyte development and is even expressed slightly earlier [4]. Using transgenic mice, it was found that Pax3 is required during ontogenesis to expand the pool of pre-melanoblasts early in development, whereas MITF facilitates survival of melanoblasts immediately after migration from the dorsal neural tube [5]. Cutaneous melanomas arise from melanocytes, mostly from the nevus-clustered pigment cells called nevocytes [6,7]. MITF is a protein determining the lineage identity of normal and malignant melanocytes. Tachibana and coworkers revealed that even exogenously expressed MITF is able to convert NIH/3T3 fibroblasts into cells with characteristics of melanocytes expressing tyrosinase and TYRP1 [8].

The MITF gene was first discovered in a mouse strain containing a transgene inserted into mouse chromosome 6 that caused a previously described phenotype described: reduced eye size, loss of skin pigmentation, defects and abnormalities in osteoclasts and mast cells and early deafness. Thus, fortuitous disruption of the MITF gene by a transgene led to the discovery of the MITF gene and protein [9]. A parallel finding confirmed the same phenotype ascribed to disrupted MITF function [10]. Thus, the loss of pigmentation and other abnormalities observed in mice for several decades [11] were clarified by cloning MITF and analyzing its mutations. Subsequently, the human MITF gene on chromosome 3 was identified and cloned. The MITF protein showed striking homology to its mouse and other species orthologs [12,13], especially within the critical functional domains. Mutations in the MITF gene cause Tietz syndrome [14] and Waardenburg syndrome, which is also caused by mutations of Pax3 or Sox10 [15,16]. Many MITF mutations have been found in both syndromes or in melanomas, and the majority of mutations prevent binding of MITF to DNA, thus disabling its function [17]. In the following studies, the melanocyte-specific isoform MITF-M (named hereafter MITF) then appeared to be a master regulator of transcription in pigment cells, a pro-survival factor for melanocytes and melanoma cells and an oncogene in melanoma. It is involved in all basic cellular processes of normal and malignant melanocytes, such as differentiation, proliferation, invasion, migration, cell survival, senescence, replication and DNA damage repair. Here we review the basic knowledge of the MITF gene and protein, and highlight the multifunctional role of MITF in cutaneous melanoma as well as questions that have not yet been fully resolved.

## 2. MITF Gene and Protein

The MITF gene harbors nine distinct promoters that initiate transcription of different first exons which are more or less specific for various cell types and tissues, while the remaining eight exons are common to all MITF isoforms [18,19,20,21,22]. Some isoforms have broader expression, while others are more restricted to specific cell types and tissues, such as mast cells, osteoclasts, heart, retinal cells and some others, besides melanocytes [20,23]. The shortest first exon occurs in the melanocyte isoform of MITF. The human melanocyte-specific MITF protein contains 419 amino acids. Of all isoforms, three may also contain an additional short sequence of six amino acids just upstream of exon 6 [19,24]. MITF belongs to the Myc superfamily of transcription factors which binds to the CANNTG motif in DNA. MITF forms a smaller subfamily together with TFE3, TFEB and TFEC transcription factors (MiT-TFE family) [25]. All proteins contain a leucine-zipper dimerization domain and can form either homodimers or heterodimers. MITF is a typical basic helix–loop–helix transcription factor that harbors a short transcription activation domain near the N-terminus [26,27]. A second activation domain, which is about 6–10 times weaker, resides near the C-terminus [28,29].

The DNA-binding specificity and transcriptional activity associated with consensus motifs were studied in greater detail [30]. MITF binds more specifically to the E-boxes CATGTG or CACATG, but the best DNA binding was observed when the CATGTG was immediately flanked at the 5’end by a T residue. The broader E-box, called the M-box (mostly AATCATGTGCTTT), has been identified in many MITF-activated downstream genes [30]. Thus, MITF displays binding specificity even among various E-boxes.

## 3. Post-Translational Modifications Regulate MITF Activity

The transcriptional activity of MITF is influenced by some post-translational modifications. The most important ones are phosphorylation, ubiquitination, SUMOylation, and acetylation. c-Kit induces MITF phosphorylation at two sites, S73 (by kinase ERK1/2) and S409 (by kinase p90 Rsk-1) [31]. These modifications upregulate the transactivation capacity of MITF but simultaneously make MITF more susceptible to ubiquitin–proteasome degradation. MITF typically appears as a double band about 9–10 kD apart on SDS-PAGE. After stimulation of c-Kit by Sl, ERK1/2 activated through the MAPK pathway downstream of c-Kit transiently phosphorylates S73 causing the attenuation or disappearance of the higher band [32]. Furthermore, a specific MEK kinase inhibitor PD98059 prevents ERK-mediated phosphorylation of MITF.

Ubiquitination destabilizes the MITF molecule and predetermines it for degradation. The ubiquitin-conjugating enzyme hUBC9 ubiquitinates MITF at K201 and hUBC9 overexpression results in MITF protein degradation. Serine 73, an important MITF phosphorylation site (above) regulates the stability of the MITF protein, since a serine to alanine mutation prevents hUBC9-mediated degradation of MITF [33]. On the other hand, the deubiquitinating enzyme USP13 protects MITF by deubiquitinating the K201 amino acid residue and therefore seems to be essential to maintain elevated MITF levels and melanoma growth [34,35]. Exogenous USP13 substantially upregulates the endogenous MITF protein level without altering MITF mRNA expression. Thus, USP13 acts through the stabilization of the MITF protein by protecting it from degradation.

Two SUMOylation sites were recognized at lysine residues K182 and K316 [36]. Interestingly, SUMOylated MITF revealed normal stability and increased the transcriptional stimulation of some target promoters (such as HIF1a), while activity of other target promoters was decreased. A germline mutation corresponding to the substitution of glutamic acid for lysine at codon 318 was identified at a higher frequency in patients with melanoma, renal cell carcinoma (RCC), or both tumors [37]. Individuals carrying this mutation exhibited an approximately fivefold increased risk of developing melanoma, RCC, or both cancers. The mutation E318K occurs within the SUMOylation consensus motif IKQE and severely impairs the SUMOylation of MITF. Thus, this SUMOylation-impairing mutation predisposes to both types of cancer. The same recurrent mutation was observed in sporadic melanoma by other authors [38].

Acetylation of MITF has recently been studied in detail [39]. MITF has been found to show a long residence time which is reduced by p300/CBP-mediated MITF acetylation at lysine K206. K206 acetylation has also decreased genome-wide MITF DNA binding thereby shifting DNA binding away from the preferred MITF consensus CATGTG toward the CACGTG motif. All MITF post-translational modifications including ubiquitination and SUMOylation sites have also been reviewed in greater detail in other papers [3,19,22,40,41].

## 4. Upstream and Downstream Transcriptional Circuitry of MITF

### 4.1. Factors Activating MITF Transcription

**Figure 1:**
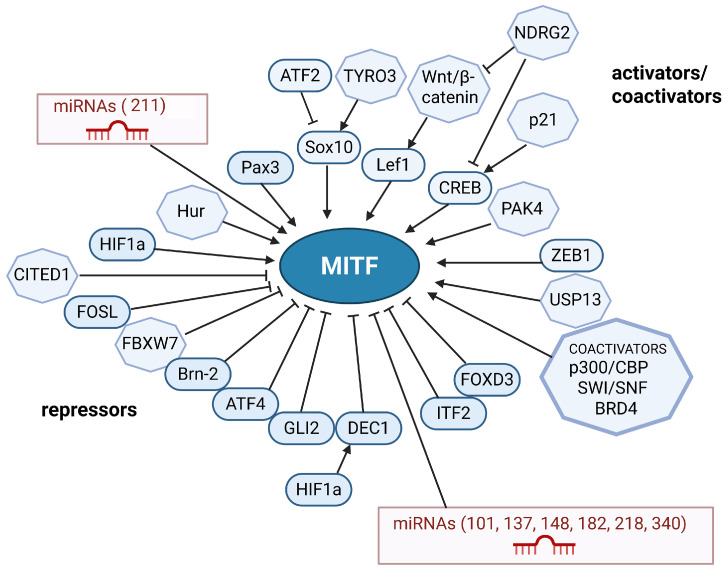
Upstream MITF activators and coactivators and proteins that repress MITF expression. Proteins which are activated and coactivated by MITF are marked by arrows; proteins that repress MITF expression are recognized by blocking bars. Please see the text for details.

Importantly, several binding sites for classical transcription factors that activate MITF gene expression (Figure 1) are clustered closely upstream of the transcription start site. Four essential transcription factors and their binding sites were identified in the proximal MITF promoter.

First, CREB mediates the canonical cAMP-PKA-CREB signaling pathway and increases activity of the MITF promoter [42,43,44,45] (Figure 1). Second, LEF1, the effector of the β-catenin/Wnt pathway, also acts as an activator of the MITF promoter [46] through the two closely located proximal binding sites. It has also been described that MITF transactivates its own promoter and interacts with LEF1, thus synergizing MITF expression [47]. MITF is downstream of the β-catenin signaling and mediates the Wnt pathway-activated proliferation of melanoma cells [48]. Moreover, MITF can interact with β-catenin and modulate the expression of MITF targets [49]. Interestingly enough, in contrast to the purely pro-oncogenic role of β-catenin in other cancers, β-catenin blocks melanoma invasion and this different role of β-catenin is mediated through MITF as it suppresses the Rho GTPase-regulated cell morphology [50]. Third, Pax3 is an important activator of MITF transcription [15,16,51,52]. Pax3 is not only the activator of MITF, but it is also important for the development of embryonic melanocytes as it expands melanoblast progenitor cells early in development, while MITF thereafter ensures the survival of melanoblasts [16]. Fourth, the Sox10 protein is a critical activator of robust MITF transcription as well, with two closely located binding sites in the MITF promoter [16]. Sox10 activity can be further potentiated by TYRO3 (a receptor protein tyrosine kinase) [53] thereby facilitating the oncogenic phenotype of melanoma cells. The Sox10 level can be downregulated by ATF2 [54]. Of note, the transcriptional activity of the Sox10 protein can be substantially influenced by SUMOylation [55]. MITF expression is downregulated by inhibiting the p21-activated kinase 4 (PAK4), indicating that also this kinase activates MITF and helps to upregulate MITF by activating LEF1 [56]. Also, LEF1 has been reported to interact with MITF directly and helps activate the MITF target TYRP2 [57]. The ZEB1 protein, involved in EMT, also possibly activates the MITF gene [58]; however, its precise role is still not entirely clear [59]. Reportedly, human antigen R (HuR) siRNA nanoparticles substantially downregulated the expression of MITF and caused G1 cell cycle arrest, demonstrating the possibility of reducing MITF expression by repressing HuR, with cellular and therapeutic consequences [60]. Some miRNAs participate in activating MITF expression (e.g., miRNAs 148 and 211); however, some other miRNAs, such as miRNAs 101,137, 148, 182, 218 and 340, repress MITF expression (reviewed in [61,62,63]).

### 4.2. MITF Coactivators

Given that CREB is an upstream positive regulator of MITF, a CREB-binding protein (CBP) or its homolog p300 protein, both of which are acetyltransferases, function as cofactors of MITF expression in the cAMP-CREB-p300 axis [27]. The strong N-terminal MITF transcriptional activation domain (TAD) is relatively short (aa. 114 to 132) and shows high homology with some regions in TFE3 and the E1A oncoprotein. As MITF and p300 can interact in vitro and in vivo [27], CBP/p300 can also participate in coactivating the various MITF targets. This was, for example, confirmed by the finding that p300/CBP coactivates MITF transcriptional activity in a manner dependent upon MITF phosphorylation as p300/CBP associates with mitogen-activated protein kinase-phosphorylated MITF [64]. Studies were also conducted to determine the coactivation by a weaker C-terminal MITF TAD. Structural studies have established that this TAD is intrinsically disordered and binds to the TAZ1 and TAZ2 domains of CBP/p300. Furthermore, the acidic motif in MITF has been found necessary for the MITF TAD-TAZ2 CBP interaction, thus contributing to the transcriptional activity of MITF [29]. Interestingly, p300 mediates the stability of the MITF activator SOX10 protein and prevents its degradation. p300 and SOX10 are also frequently co-amplified in melanomas [65] and thus contribute to the oncogenicity of melanoma cells. The ZEB2 protein is important for melanoblast differentiation and migration and probably also activates MITF expression [58]. Interestingly, the p21 protein, a known target gene of MITF and a cell cycle inhibitor, was found to be partially localized in the nucleus and to help coactivate CREB-mediated transcriptional activation of MITF [66]. In these experiments, p21 co-occupied the MITF promoter. Additionally, p21 knockdown resulted in a decrease in MITF protein level and promoter activity. p21 protein levels correlated with MITF mRNA in most cell lines tested.

SWI/SNF is a chromatin-remodeling complex containing alternative ATPases Brg1 or Brm. This complex was recognized to be essential for the transcription of several MITF target genes [67,68] and bound to the MITF protein at MITF target promoters. Either Brg1 or Brm must be present in the complex to promote expression of distinct MITF target genes and melanoma growth and tumorigenicity [69]. The expression of Brg1 increased during melanoma progression and was high in metastases. The study identified Brg1 target genes that play an important role in melanoma metastasis and showed that Brg1 promoted the invasive ability of melanoma in vitro [70]. It was further shown that the BAF60 subunit of SWI/SNF facilitated the interaction of MITF with Brg1. BAF60 (all three isoforms A, B, and C) also physically interacted with MITF and BAF60 was required for the expression of MITF target genes in differentiating developing melanoblasts [71], further suggesting the importance of SWI/SNF for melanocyte differentiation. Brg1 protected melanoma cells against UV-induced death and prevented apoptosis in UV-irradiated melanoma cells by activating the expression of the melanoma inhibitor of apoptosis, livin (ML-IAP) [72], which is an MITF-activated target gene. Importantly, SWI/SNF is pivotal for the transcription of MITF itself [73]. The Brg1 ATPase-containing complex seems to be more important for MITF expression. Chromatin immunoprecipitation confirmed the binding of Brg1 or Brm to the MITF promoter. Furthermore, knockdown of Brg1 severely compromised MITF expression with a concomitant downregulation of MITF targets and decreased proliferation of melanoma cells. Although Brm was able to substitute for Brg1 in limited melanoma cell growth and to maintain MITF expression, sequential knockdown of both Brg1 and Brm completely abolished proliferation [73]. Two other contributions confirmed the importance of Brg1. Brg1 was essential for melanoma cell proliferation in vitro and for normal melanocyte development in vivo and helped MITF to actively regulate many target genes [74,75]. Furthermore, increased proliferation of melanoma cells required a functional SWI/SNF complex not only by supporting the expression of MITF and its targets and but also by activating the expression of some pro-survival proteins not directly regulated by MITF [76].

The acetylhistone reader and chromatin-remodeling protein Brd4 (a member of the BET family) is another recognized epigenetic coactivator in melanoma and an accelerator of its growth. BRD4 and BRD2 were amplified in a subset of melanomas and BRD4 was overexpressed especially in metastases [77]. The growth of melanoma cells was significantly impaired in the presence of BET inhibitors and a number of typical pro-oncogenic proteins was downregulated, together with upregulation of cell cycle inhibitors p21 and p27 [77], independently of BRAF or NRAS mutations. The data represent BRD4 as a potential therapeutic target. Biton and colleagues have recently specified the action of BRD4 in detail. The lysine methyltransferase SETD6 methylates Brd4 at lysine 99 in melanoma cells and the SETD6 knockout or a point mutation of Brd4-K99 disrupts Brd4 occupancy at the MITF promoter [78]. This clearly shows the importance of the recruitment of BRD4 and MITF to melanoma cell chromatin. Other studies also pointed out the importance of the Brd4 protein as a cofactor of MITF in the MITF-mediated transcription of its target genes [79]. Both Brd4 and Brd2 interacted with MITF and were detected near the MITF-binding sites in the target promoters and accelerated the binding of MITF to DNA. Taken together, the data implicate the BET family of proteins in the regulation of melanocyte differentiation as MITF coactivators [79].

### 4.3. Factors Repressing MITF Transcription

MITF can activate or repress various genes resulting in specific phenotypes. While many factors activate and coactivate MITF expression (above), several proteins have been reported to repress MITF transcription. Brn-2 was reported to be a repressor of MITF transcription [80] and bound directly to the MITF promoter [81]. Reciprocal expression between Brn-2 and MITF in mouse xenografts was observed [82]. An inverse correlation between MITF and Brn-2 expression [83] and their inverse expression during melanoma invasiveness were observed. In particular, Brn-2 directly induced tumor suppressor PTEN expression whereas MITF repressed PTEN transcription [84]. The invasiveness of Brn-2 was ascribed to its cooperation with NOTCH1/2 signaling and to the importance of the EZH2 methyltransferase through which this invasiveness is maintained [85]. The effect of Brn-2 on melanoma cell proliferation is also regulated by β-catenin [86].

The HIF1a protein is also a suppressor of MITF through the DEC1 factor. Hypoxic conditions, which are less favorable for melanoma growth, can be rescued by cotransfection of MITF [87]. Inactivation of the tumor suppressor FBXW7, whose loss or mutation is observed in melanomas and whose inactivation leads to NOTCH1 activation [88], causes elevated MITF in melanoma cells. It implies that this factor also negatively regulates the MITF level [89]. Activated NOTCH1 signaling has also been reported to downregulate the MITF transcription [90]. The AP-1 transcription factor is another candidate for MITF repression, as AP-1 is present in low-MITF populations of tumor cells, which are slow cycling and invasive [91]. ATF4, whose production increases in response to high reactive oxygen species, suppresses MITF expression by competing with CREB [92]. Negative correlation was reported between the expression of GLI2 and MITF in melanoma cells; however, it is unclear whether MITF is directly transcriptionally repressed by GLI2 [93,94]. GLI2 plays a role in melanoma invasion and BRAF inhibitor resistance, suggesting its possible participation in the downregulation of MITF.

Some other factors such as ITF2 and FOXD3 were also implicated in MITF repression [95]. Both melanocytes and glial cells are derived from the neural crest. FOXD3, a transcriptional repressor, is expressed only in neural/glial precursors and MITF is expressed in melanoblasts. Exogenous expression of FOXD3 was found to repress MITF [96]. Further, it was observed that loss of MITF in melanoblasts causes them to transdifferentiate to glial cells. FOXD3 did not bind directly to the MITF promoter, but interacted with the PAX3 protein to prevent the binding of PAX3 (an MITF transcriptional activator) to the MITF promoter. NDRG2, a possible tumor suppressor which downregulates N-myc, was suggested to act as an MITF repressor by preventing cAMP- and β-catenin-mediated repression of the MITF promoter [97] in mouse melanoma B16F10 cells.

## 5. MITF-Regulated Genes

### 5.1. Genes Activated by MITF

**Figure 2:**
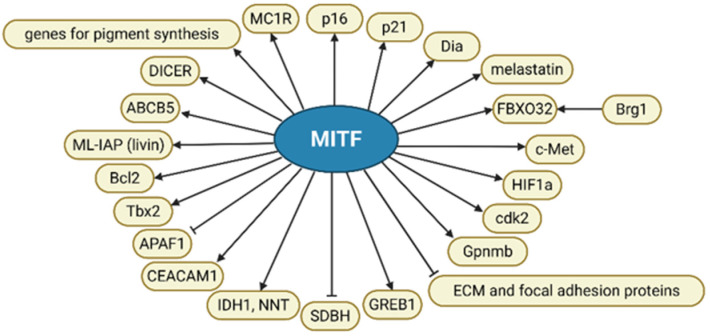
Schematic view of the main genes transcriptionally activated (or repressed) by MITF. Proteins which are activated by MITF are marked by arrows; proteins that are repressed are marked by blocking bars. Please see the text for details.

The first recognized MITF targets were the three important enzymes for melanin synthesis, tyrosinase and tyrosinase-related proteins 1 and 2 (TYRP1 and TYRP2) [43,44] (Figure 2). The tyrosinase promoter requires only about 115 proximal nucleotides including the initiator (several bases upstream of the transcription start site) [98]. While these proteins are all expressed in normal melanocytes, some pigment-forming enzymes, especially TYRP1, can often be incompletely expressed in melanoma cells in vitro, even if the MITF expression is normal [99,100,101]. This suggests that MITF is insufficient for their expression in some melanoma cell lines.

Apart from the first three melanin-synthetizing enzymes, MITF activates virtually all genes important for the biosynthesis of the pigment melanin and its transport into keratinocytes (reviewed in [61]). MITF targets also include SILV and MLANA genes, which are frequently expressed in pigment cells. MITF upregulated the activity of their promoter regions in reporter experiments. Both genes contain conserved MITF DNA motifs that are bound by MITF in vitro and in vivo [102]. A large number of novel MITF targets were identified by a two-step DNA microarray, many of which were new MITF-regulated genes [103]. In addition, MITF binds and transactivates the MC1R promoter (involved in CREB-cAMP signaling), thus creating a positive feedback loop [104].

It is intriguing that MITF activates many pro-oncogenic and antiapoptotic genes, but also antioncogenic genes or typical tumor suppressors. This may be explained by context-dependent expression (below). The HIF1a protein, which was reported to repress MITF expression through the DEC1 protein [87], has also been shown to be activated by MITF through the cAMP-CREB-mediated pathway [105]. It has been demonstrated that MITF also bound to the HIF1a promoter and stimulated its transcriptional activity, thus playing a pro-survival role in melanoma cells.

The c-Met promoter is bound by MITF and disruption of MITF blocks HGF-dependent increases in endogenous c-Met, implicating c-Met as another MITF target [106]. Tbx2, a melanoma pro-proliferative and antisenescence protein [107], has been discovered to be a typical MITF target with MITF-binding elements in its promoter, thus allowing direct activation by MITF [108]. Tbx2 may also be regulated by Pax3, an MITF activator [109]. PAX3 was shown to transcriptionally activate Brn-2, leading to direct repression of MITF expression [110]. FBXO32, a component of the SCF E3 ligase complex, is a newly recognized MITF target gene which promotes melanoma migration and proliferation. Furthermore, Brg1 is a coactivator of FBXO32 transcription [111]. The Ig-like adhesion molecule (CEACAM1), overexpressed in many cancers, is elevated in melanoma as well and its expression correlates with upregulated MITF, which has binding sites in the CEACAM1 promoter and MITF acts as a direct activator of CEACAM1. So, CEACAM1 contributes to melanoma progression through MITF [112]. The GREB1 gene is involved in the progression of some tumors. Its splicing variant Is4 which encodes the C-terminal half of GREB1 was described to be upregulated in melanoma. The MITF-binding motifs necessary for GREB1 transcription were localized to exon 19. Moreover, GREB1 Is4 silencing reduced melanoma cell proliferation [113], implying GREB1 Is4 in melanoma development. The Gpnmb protein was sufficient to drive expression of melanoblasts in vivo and also appears to be positively regulated by MITF [114].

Importantly, MITF directly targets the Dia promoter and increases its transcription. The Dia1 protein promotes actin polymerization and regulates Skp2 kinase which accelerates proliferation and degradation of the p27 cell cycle inhibitor. A rheostat model has been proposed in which high MITF expression is present in non-invasive differentiated cells and low MITF marks invasive subpopulations (see below). ABCB5 is a marker of melanoma-initiating cells [115] and is linked to drug resistance. ABCB5 is a direct target of MITF and is induced by β-catenin that also coactivates MITF expression. It has been suggested that the ABCB5 protein may mark a slow-cycling but differentiated population of melanoma cells [116].

MITF also regulates genes that protect melanoma cells from reactive oxygen species (ROS). Through some of its direct targets such as IDH1 and NNT, MITF becomes a significant driver of the cellular antioxidant state [117]. Other genes that are typical tumor suppressors are directly regulated by MITF. Melastatin (TRPM1), which correlates with a better melanoma prognosis, is a direct target of MITF transcription. It contains three MITF-binding sites in the promoter, the activity of which increases sharply in response to exogenous MITF. Melastatin expression is also linked to melanoma differentiation [118]. Remarkably, a promoter of the DICER gene, which codes a miRNA processing protein, also joins the repertoire of MITF target genes [119].

### 5.2. Genes Repressed by MITF

Dishat and coworkers showed that MITF directly repressed the expression of proteins involved in EMT and focal adhesion, such as cadherin 2 [120]. MITF knockdown increased focal adhesion and drug resistance in melanoma cells. It has also been observed that MITF can repress its own expression and regulates SDHB to control the TCA cycle and suppresses pseudo-hypoxia [121].

## 6. MITF as an Oncogene and Marker in Melanoma

### 6.1. MITF Is an Antiapoptotic Factor and Melanoma Oncogene

MITF maintains the identity of melanoma cells and acts as a critical antiapoptotic and pro-proliferative protein. It is indisputable that MITF is absolutely required for the progression of melanoma to later stages [18,63,122,123,124]. MITF is regarded as a pro-survival and lineage-determining factor in pigment cells [3,18,62,63,122,123,124]. The MITF gene is amplified in a subset of melanomas that correlates with decreased patient survival. Thus MITF has been regarded as a lineage-addiction oncogene in melanoma, which is required for tissue-specific cancer development [125]. MITF negatively regulates the APAF1 gene thereby increasing resistance to MAPK inhibitors [126]. APAF1 protein expression is frequently lost in melanomas [127]. The absence of this important proapoptotic cell-death effector which cooperates with cytochrome c and caspase-9 renders APAF1-negative melanomas chemoresistant and unable to accomplish the apoptotic program.

Bcl-2 protein plays a pivotal role among the antiapoptotic factors in melanoma. Its gene is directly transcriptionally activated by MITF. The linkage between MITF and Bcl-2 helps explain the essential roles of both proteins in the melanocyte lineage survival. Germline deletion of Bcl-2 results in melanocyte loss and exhibits a phenotype similar to that with MITF mutations [122]. BCL2A1, another Bcl-2 family member, has been shown to be amplified in about one-third of melanomas [128]. ML-IAP (livin) is an antiapoptotic protein expressed in melanoma. MITF binds the ML-IAP promoter, which is then strongly activated by MITF. Depletion of MITF by siRNA leads to a reduction in melanoma cell viability, which could be rescued by ectopic expression of ML-IAP, implicating MITF and ML-IAP in cell survival [129]. The contribution of HIF1a to the survival of melanoma cells has been mentioned above [87].

Importantly, although MITF transcriptionally activates the cell cycle inhibitors p16 and p21 (below), it also acts as a bona fide activator of the critical cell cycle regulator cdk2 through binding to the cdk2 promoter in melanoma cells but not in non-melanoma tumor cells [130]. Low MITF expression correlates with low cdk2 in melanoma cell lines and in primary melanoma specimens. Furthermore, cdk2 downregulation suppressed the growth of melanoma cells. Thus, cdk2 is a critical MITF target that mediates its pro-proliferative function [130].

### 6.2. MITF Is a Specific Melanoma Marker

MITF is invariably expressed at least in the initial melanoma stages whereas invasive populations and metastases may eventually lose its expression (below). MITF plays a central role in melanoma survival and differentiation. Given that MITF is reliably present in skin, nevus and melanoma cells, it has long been recognized as an excellent marker for melanocytic lesions [131]. MITF is even more reliable than HMB-45, S-100 or Melan-A proteins commonly used as immunohistochemical melanoma markers. MITF is localized to the nucleus and because the diagnosis of malignant melanoma can sometimes be challenging, nuclear MITF is often the most important marker in routine immunohistochemical analysis. It can help discriminate rare desmoplastic variants of melanoma which can mimic non-melanocytic tumors such as neurofibromas. MITF is also present as a marker in melanocytic clear cell sarcomas of soft tissues [132]. MITF was found to be highly specific for melanoma diagnosis, including amelanotic and lentigo maligna melanoma, both of which were frequently stained with an MITF-specific antibody. Micrometastases in sentinel lymph nodes were also positively stained [133]. The antibody used primarily for MITF detection, D5, can also detect isoforms other than the melanocyte-specific one; however, the staining is typically weaker and cytoplasmic. In cytological specimens, all 44 melanoma samples, including desmoplastic, stained positively for MITF, whereas only one of 37 non-melanocyte tumor samples was positive [134]. Although a subset of melanomas, particularly desmoplastic and spindle cell melanomas, still remain diagnostically challenging, immunohistochemical detection of MITF has become a routine and reliable method to detect cells of melanocytic origin. In some cases, HMB-45, Melan-A and S-100 protein could help to determine diagnosis [135,136]. MITF was reported to be a valuable marker in three melanoma cases, with greater specificity than other markers [137]. Taken together, the melanocyte-specific isoform of MITF appears to be a unique molecule in the differential diagnosis of melanocytic tumors.

## 7. The Rheostat Model for MITF

Importantly, Carreira et al. have revealed that MITF directly targets the DIAPH1 promoter and activates its transcription. The Dia1 protein promotes actin polymerization and coordinates actin and microtubule networks. It also regulates Skp2 kinase that accelerates cell proliferation. Skp2 is involved in the degradation of the cell cycle inhibitor p27/KIP [138] and in cell proliferation. Based on these findings, a rheostat model has been coined first by the C. Goding group [138], suggesting that high MITF expression upregulates Dia1, inhibits invasiveness and supports proliferation of melanoma cells. Low MITF and low Dia1 lead to p27 downregulation by its accelerated degradation by Skp2, accompanied by a phenotype with slow proliferation and increased invasiveness. Thus, low-MITF levels are associated with highly invasive cell populations whereas high-MITF levels support differentiation and suppress invasiveness.

Several subsequent reports described similar results. Heterogeneity in tumor subpopulations represents the major obstacle to effective cancer treatment. In melanoma, populations of cells with stemness properties (melanoma-initiating cells) proliferate slowly but exhibit high metastatic potential and resistance to therapy. In a B16 mouse melanoma model, although the high-MITF level promoted melanoma cell growth, low-MITF or MITF-depleted cells were slow-growing but still displayed high tumorigenic potential and increased expression of the stem cell markers Oct4 nad Nanog [139]. Ablation of this cell population dramatically decreased tumor formation. These results support the postulated rheostat model.

Although PAX3 itself does not play a substantial role in melanoma cell proliferation, knockdown of PAX3 inhibits cell migration in lower-MITF melanoma cell lines. In contrast, MITF knockdown promotes cell migration in higher-MITF melanoma cell lines [140], supporting the role of PAX3 in melanoma phenotype switching. The MAPK pathway is frequently activated in melanomas. Consequently, the expression of the oncogenic transcription factor FOSL1 increases, reinforcing the activity of the oncogenic factors myc, E2F3 and AP-1. At the same time, FOSL1 downregulates MITF and upregulates expression of the oncogenic kinase AXL [141]. Howlin et al. additionally reported the repression of the MITF promoter by CITED1, a transcriptional co-regulator expressed in low-MITF populations. Whole-genome expression analysis further identified a phenotype shift induced by CITED1 silencing, primarily driven by altered expression of MITF and its target genes [142].

Many results supporting the rheostat model have since been published. Lack of MITF expression was associated with resistance to targeted inhibitors. In inhibitor-resistant cells, MITF levels were inversely correlated with the expression of several oncogenic receptor tyrosine kinases, most frequently AXL. The MITF-low/AXL-high/drug-resistance phenotype has become established as highly invasive and prometastatic. The AXL inhibitor enhanced melanoma cell elimination through BRAF or ERK inhibition [143,144]. Melanomas exhibit high plasticity, enabling switching between subpopulations with different phenotypes. Proliferative melanoma cells can switch to an invasive slow-cycling state in which MITF is downregulated, leading to acquired drug resistance [145]. Currently, it is generally accepted that the inverse relationship between MITF and the tyrosine kinase AXL is the most useful marker for assessing invasive slow-proliferating populations (high-AXL/low-MITF cells) [146].

Spontaneous decrease in MITF expression is inevitably accompanied by other changes upstream of MITF (activators and coactivators). To determine the effects of downregulating MITF expression while maintaining the expression of other proteins, MITF was inducibly downregulated in five melanoma cell lines using a lentiviral shRNA system. Surprisingly, MITF knockdown caused only moderate attenuation of melanoma cell proliferation. Concomitantly, downstream differentiation markers and the known MITF targets melastatin and tyrosinase were profoundly decreased. After the MITF decline, invasiveness was not appreciably affected. These data proposed that MITF downregulation alone does not appreciably affect proliferation or phenotype switching but reduces differentiation of melanoma cells. Consequently, it has been suggested that MITF decrease or loss may accompany, rather than directly cause, the invasiveness of melanoma cells [147].

## 8. Contradictory Roles of MITF in Melanocytes and Future Prospects

Intriguingly and significantly, typical tumor suppressors are transcriptional targets of MITF. First, MITF directly activates the promoter of the cell cycle inhibitor p16 protein (INK4A) in melanoma cells and normal melanocytes. Thus, MITF can regulate the p16-Rb pathway and slow the cell cycle, possibly depending on the cellular context [148]. Notably, INK4A is mutated in about 40% of melanomas and in benign nevi as well. Second, MITF also activates transcription of another cell cycle inhibitor, p21 (CDKN1A) [149], which can induce cell cycle arrest. These distinct MITF functions are likely context-dependent. MITF can also induce Tbx2 expression while Tbx2 can both repress the p21 promoter and suppress senescence. This mechanism may help explain the Tbx2 contribution to the pro-proliferative effects of MITF in a specific cellular context. Moreover, cooperation between MITF and retinoblastoma protein enhanced the MITF-mediated activation of p21 transcription [149,150].

Somewhat divergent data were published on the expression of p16 and p21 and defects in their genes in nevi and in tumors. Deletions of INK4A (p16) were found in four out of six primary and six out of 14 metastatic melanoma cell lines but not in three metastatic melanoma specimens. INK4A deletions were detected in all eight benign nevi examined. In contrast to p16, expression of p21 was detected in all three melanocyte specimens, in all three benign nevi, and in more than half of malignant melanoma cell lines and specimens [151]. Another study revealed a high expression of p16 protein in benign nevi and a gradual loss of p16 expression level in advanced lesions [152]. It has been suggested that benign nevi, arrested in oncogene-induced senescence, express high levels of the p16/INK4A and p14ARF tumor suppressors. However, other data have shown that INK4A/ARF-encoded proteins can be dispensable for this senescence program in which the growth of nevus cells is halted [153]. As described above, some issues concerning the MITF activity remain to be clarified. In particular, MITF is capable of activating many pro-oncogenic genes, while at the same time acting as a transactivator of cell cycle inhibitors, such as p21/KIP or p16/INK4A. This may depend on the cellular context, specific features of melanoma cell lines, authentic melanomas, or normal melanocytes, which may differ in their susceptibility to activation of these genes.

**Figure 3:**
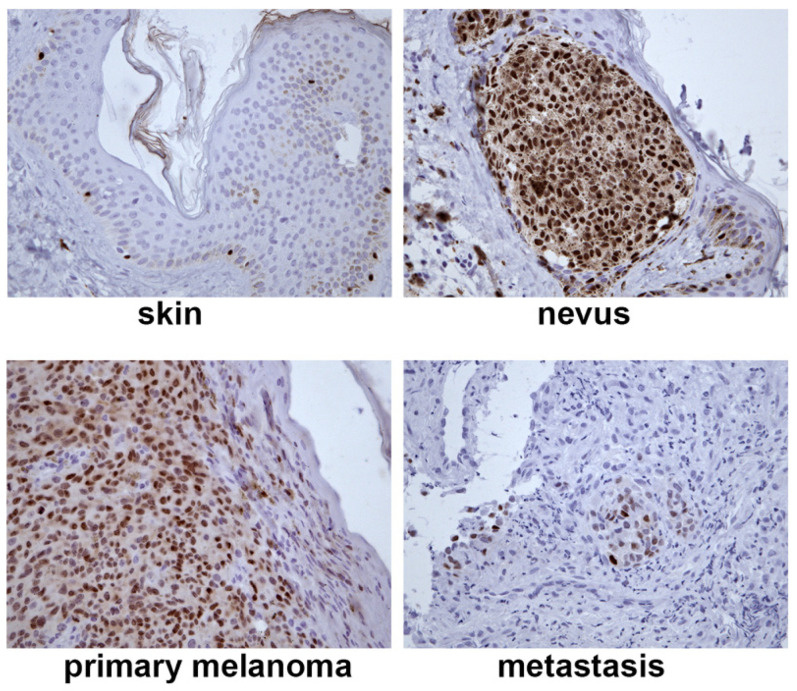
Staining of MITF in the skin, nevus, primary melanoma, and melanoma metastasis. Immunohistochemical staining of paraffin sections was performed with the anti-MITF antibody D5. Only a minor fraction of metastatic melanoma cells are stained with the anti-MITF antibody (200x).

Next, so-called “amelanotic” melanomas might have lost MITF expression or may still retain it but the pigment is not formed due to some downstream defects. If MITF expression is completely lost in late stages of melanomas such as in metastases (Figure 3), some other antiapoptotic proteins must serve as surrogates to maintain tumor growth. Although this question is still not entirely clear and different proteins may substitute the lost MITF function, a strong candidate is the oncogenic AXL kinase present in invasive populations. The melanomas without MITF expression resemble undifferentiated tumors rather than melanomas (Figure 3).

In addition, two melanoma cell lines lacking expression of MITF and its downstream targets, tyrosinase, TYRP1 and TYRP2, were found not to be induced to restore the melanotic phenotype. Expression of exogenous MITF did not lead to re-expression of the tyrosinase and TYRP1 targets [100]. Thus, when the expression of MITF was absent in two human melanoma cell lines, the melanogenic phenotype was not restored after the transfer of an exogenous MITF gene. Ectopic MITF was normally expressed in the nucleus but the transcription of the three targets could not be re-established. So, endogenous promoters in these melanoma cells became unresponsive to the properly expressed ectopic MITF. This suggests that a specific nuclear context, as yet unclear, is required for MITF to activate the melanocyte markers in malignant melanocytes and this specificity is lost concomitantly with the loss of MITF.

It is unknown how MITF could control the immune response. Pioneering work has provided the first insight into this issue. By controlling melanoma recognition by NK-cells MITF can regulate the melanoma response to the innate immune system through ADAM10 [154]. Furthermore, a correlation has been observed between PD-L1 and MITF protein expression, implicating MITF as a potential activator of PD-L1 [155]. Clearly, more data are needed to understand the role of MITF in the antitumoral immune response.

## 9. Conclusions

Melanoma is a highly relentless and drug-resistant tumor. It expresses MITF, a major transcription factor functioning as a lineage identity factor, a pro-survival and pro-proliferative protein. It is regarded as a melanoma oncogene. MITF integrates diverse microenvironmental cues to coordinate cell survival, escape from senescence, differentiation, proliferation, invasion, replication, and DNA damage repair. The plasticity of the microenvironment enables switching between two main phenotypes, invasive slow-growing low-MITF and proliferated fast-growing high-MITF cell populations. MITF activates several proteins with apparently antagonistic functions.

## Data Availability

No new data were created or analyzed in this study.

## Abbreviations

The following abbreviations are used in this manuscript:

MITF	Microphthalmia-associated transcription factor
MITF-M	melanocyte-specific isoform of MITF
CREB	cAMP responsive element-binding protein
PAX3	paired box 3
SOX10	SRY-box transcription factor 10
LEF1	lymphoid enhancer binding factor 1
Brn-2	POU class 3 homeobox 2
ATF4	activating transcription factor 4
ATF2	activating transcription factor 2
Dia1	diaphanous-related formin 1 (DIAPH1)
BPTF	bromodomain PHD finger transcription factor
BRD4	bromodomain containing 4
SOX2	SRY-box transcription factor 2
HIF1a	hypoxia-inducible factor 1 subunit alpha
p300	EP300 lysine acetyltransferase
CBP	CREB-binding protein (lysine acetyltransferase)
Cdk2	cyclin-dependent kinase 2
EZH2	enhancer of zeste 2 polycomb repressive complex 2 subunit
Oct4	POU class 5 homeobox 1 (POU5F1)
DICER	dicer 1, ribonuclease III
Tbx2	T-box transcription factor 2
ZEB1	zinc finger E-box binding homeobox 1
ZEB2	zinc finger E-box binding homeobox 2
PPAR	peroxisome proliferator-activated receptor alpha
SNAIL1	snail family transcriptional repressor 1
SNAIL2	snail family transcriptional repressor 2 (SLUG)
AXL	AXL receptor tyrosine kinase
MC1R	melanocortin 1 receptor
Notch	notch receptor 1
TRPM1	transient receptor potential cation channel subfamily M member 1
FOSL1	FOS-like 1, AP-1 transcription factor subunit
HMGA1	high mobility group AT-hook 1
FBXW7	F-box and WD repeat domain containing 7
FBXO32	F-box protein 32
HMB-45	premelanosome protein, PMEL
MLANA	melan-A
SETD6	SET domain containing 6, protein lysine methyltransferase
Gpnmb	glycoprotein nmb
STAT3	signal transducer and activator of transcription 3
YY1	YY1 transcription factor
IRF4	interferon regulatory factor 4
TFAP2A	transcription factor AP-2 alpha
PAK4	p21 (RAC1)-activated kinase 4
Wnt	wingless
GREB1	growth-regulating estrogen receptor binding 1
JUN	Jun proto-oncogene, AP-1 transcription factor subunit
TFE3	transcription factor binding to IGHM enhancer 3
TFEB	transcription factor EB
TFEC	transcription factor EC
Sl	steel factor (c-kit ligand)
c-Kit	KIT proto-oncogene, receptor tyrosine kinase
MAPK	mitogen-activated protein kinase
c-MET	proto-oncogene, receptor tyrosine kinase
HGF	hepatocyte growth factor
Rsk-1	ribosomal S6 kinase 1
USP13	ubiquitin-specific protease 13
SUMO	small ubiquitin-like modifier
FOXM1	forkhead box M1
FOXD3	forkhead box D3
TAD	transactivation domain
IDH1	isocitrate dehydrogenase (NADP(+)) 1
NNT	nicotinamide nucleotide transhydrogenase
BET	bromodomains and extra-terminal
BRG1	SWI/SNF-related BAF chromatin-remodeling complex subunit ATPase 4
BRM	SWI/SNF-related BAF chromatin-remodeling complex subunit ATPase 2
ML-IAP	baculoviral IAP repeat containing 7 (livin)
CEACAM1	CEA cell adhesion molecule 1
ITF2	transcription factor 4
ABCB5	ATP-binding cassette subfamily B member 5
CITED1	Cbp/p300 interacting transactivator with Glu/Asp rich carboxy-terminal domain 1
NDRG2	N-myc downstream-regulated gene 2
EMT	epithelial-to-mesenchymal transition
SDHB	succinate dehydrogenase subunit B
APAF1	apoptotic peptidase activating factor 1
INK4A	cyclin-dependent kinase inhibitor 2A
ADAM10	ADAM metallopeptidase domain 10
TYRP1	tyrosinase-related protein 1
TYRP2	tyrosinase-related protein 2
CDKN1A	cyclin-dependent kinase inhibitor 1A (p21)
